# Fidaxomicin for the Treatment of *Clostridioides difficile* Infection in Adult Patients: An Update on Results from Randomized Controlled Trials

**DOI:** 10.3390/antibiotics11101365

**Published:** 2022-10-06

**Authors:** Daniele Roberto Giacobbe, Antonio Vena, Marco Falcone, Francesco Menichetti, Matteo Bassetti

**Affiliations:** 1 Department of Health Sciences (DISSAL), University of Genoa, 16132 Genoa, Italy; 2 Infectious Diseases Unit, IRCCS Ospedale Policlinico San Martino, 16132 Genoa, Italy; 3 Infectious Diseases Unit, Department of Clinical and Experimental Medicine, Azienda Ospedaliera Universitaria Pisana, University of Pisa, 56126 Pisa, Italy

**Keywords:** fidaxomicin, CDI, rCDI, *Clostridioides difficile*, randomized clinical trials, RCT

## Abstract

In recently updated international guidelines, fidaxomicin is preferentially recommended as first-line treatment over vancomycin both for the first episode of CDI and for rCDI, based on the results of different randomized controlled trials (RCTs). Although noninferiority was the rule in phase-3 RCTs with regard to the primary endpoint of clinical cure, for shaping these recommendations, particular attention was devoted to the improved global cure and reduced risk of recurrent CDI (rCDI) observed with fidaxomicin compared to vancomycin in RCTs. Overall, while the major driver of choice should remain the global benefit for the patient, consideration of available resources should be necessarily weighed in the balance, since fidaxomicin still remains more costly than vancomycin. Against this background, precisely stratifying risk groups for rCDI will represent a crucial research trajectory of future real-life studies on the treatment of first CDI episodes. In the current narrative review, we discuss the updated evidence from RCTs on the efficacy of fidaxomicin for the treatment of either the first CDI episode or rCDI, which eventually supports its positioning within current treatment algorithms and guidelines.

## 1. Introduction

*Clostridioides difficile* is the most common causative agent of infectious diarrhea in hospitalized patients; although, community-acquired *C. difficile* infection (CDI) has also become epidemiologically and clinically relevant during the last decade [1,2,3,4,5,6].

In the treatment approach to CDI, clinicians aim both to cure the index episode and to reduce the risk of recurrences. Indeed, recurrent CDI (rCDI) develops in 10–30% of cases after the first CDI episode, with the risk further increasing with each successive episode [7,8,9,10]. In the recently released updates of guidelines/guidance documents from the Infectious Diseases Society of America/Society for Healthcare Epidemiology of America (IDSA/SHEA) and from the European Society of Clinical Microbiology and Infectious Diseases (ESCMID), there have been changes in the recommendations pertaining to the use of fidaxomicin, a macrocyclic antibiotic approved both in the US and in Europe for the treatment of CDI [11,12].

In the present narrative review, we discuss the updated evidence from randomized controlled trials (RCTs) on the efficacy of fidaxomicin for the treatment of either the first CDI episode or rCDI, which eventually supports its positioning within current treatment algorithms and guidelines.

## 2. Methods

In August 2022, we performed a PubMed search using the keyword “fidaxomicin”. After title and abstract screening of the retrieved 657 records, 227 of them were selected for initial full-text assessment. In line with the narrative nature of the present review, relevant articles pertaining to the topic were further selected by the authors and organized in the following structure: (i) an introductory section on the characteristics, mechanism of action, and antimicrobial activity of fidaxomicin; (ii) a main section on the results from phase-3/4 RCTs; (iii) a conclusions section.

## 3. Characteristics, Mechanism of Action, and Antimicrobial Activity of Fidaxomicin

Fidaxomicin, administered orally, is the first member of the macrocycles class of antibiotics, and it shows bactericidal activity against *C. difficile* [13,14]. In addition, fidaxomicin has negligible activity against other bacteria constituting the gut microbiota [15,16]. This selective activity relies on the fact that the *C. difficile* RNA polymerase (inhibited by fidaxomicin [17]) has a specific residue (lysine 84) that is bound by fidaxomicin and acts as a crucial sensitizer allowing fidaxomicin killing activity [18]. This specific residue is absent in gut bacteria belonging to the phyla Bacteroides and Proteobacteria [18,19]. In line with the largely reported more favorable effect than other CDI treatments in terms of microbiota disruption [16,20,21,22,23], combined with its modest activity (although inhibitory at the achieved stool concentrations) against vancomycin-resistant enterococci (VRE) [24], fidaxomicin treatment resulted in a reduced frequency of novel stool culture positivity for vancomycin-resistant enterococci (VRE) and *Candida* spp. compared to vancomycin among patients with negative pre-treatment stool cultures enrolled in a phase-3 RCT (7% vs. 31% for VRE acquisition among 247 patients, *p* < 0.001; 19% vs. 29% for *Candida* spp. acquisition among 252 patients, *p* = 0.03) [25]. In patients with pre-treatment VRE colonization, a larger decrease in the mean stool concentration of VRE was observed with fidaxomicin therapy than with vancomycin therapy; although, selection of some subpopulations of VRE with high fidaxomicin minimum inhibitory concentration (MIC) was observed during fidaxomicin treatment [25]. Of note, in patients receiving vancomycin, the risk of colonization and subsequent bloodstream infections by *Candida* spp. or enterococci may be possibly higher among those receiving high vancomycin dosages (>500 mg/day) [26].

Another peculiar characteristic of fidaxomicin, not shared by other anti-*C. difficile* agents such as vancomycin and metronidazole, is its long post-antibiotic effect, which might be relevant considering the hastened intestinal transit and drug elimination in patients with diarrhea [14,27]. Following oral administration, fidaxomicin is poorly absorbed, reaching high intracolonic concentrations [28,29]. Together with the lack of cytochrome P450 metabolism, the very low bioavailability of fidaxomicin may explain its low potential for systemic adverse events and drug interactions [14,30,31]. The main metabolite of fidaxomicin, OP-1118, is produced in vivo by the action of an esterase, and retains antimicrobial activity against *C. difficile* [13,32,33].

The activity of fidaxomicin against *C. difficile* has been assessed in several in vitro studies. Among 403 non duplicate *C. difficile* isolates from Taiwan, fidaxomicin showed potent in vitro activity, with MIC_90_ of 0.5 mg/L (range ≤ 0.015 to 0.5 mg/L) [34]. An even lower MIC_90_ of 0.125 mg/L was measured among 188 *C. difficile* isolates from Hungary, with only four isolates displaying a MIC value of 0.5 mg/L [35]. In another surveillance study on 925 *C. difficile* isolates from the US, MIC_90_ for fidaxomicin was 0.5 mg/L, with a range from 0.004 to 4 mg/L [36]. The same MIC_90_ of 0.5 mg/L, with a range from 0.004 to 1 mg/L, was observed in a subsequent update on a larger sample of 1889 *C. difficile* isolates [37]. In a surveillance study from Japan, MIC_90_ for fidaxomicin was 0.25 mg/L among 100 *C. difficile* isolates (range 0.03 to 0.5 mg/L) [38]. A MIC_90_ of 0.25 mg/L for fidaxomicin was also observed in a surveillance study on 105 *C. difficile* isolates from Thailand (range 0.004 to 0.25 mg/L) [39]. Among 101 *C. difficile* isolates from China, MIC_90_ for fidaxomicin was 0.5 mg/L (range 0.032 to 1 mg/L), whereas it was 0.03 mg/L among 100 *C. difficile* isolates from the US in another study (range ≤ 0.008 to 8 mg/L) [40,41]. In a small study on 64 *C.* *difficile* isolates from the Czech Republic, MIC_90_ for fidaxomicin was 0.125 mg/L (range 0.06 to 0.25 mg/L) [42]. Fidaxomicin showed the greatest in vitro potency compared to the other seven antimicrobial agents tested against 1310 *C. difficile* isolates from Canada (with 027 being the most frequent ribotype, 24.5%), showing a MIC_90_ of 0.25 mg/L (range 0.055 to 2 mg/L) [43]. In a large pan-European surveillance study of 953 *C. difficile* isolates, MIC_90_ for fidaxomicin was 0.125 mg/L (range ≤ 0.002 to 0.25 mg/L), and all strains were considered susceptible according to an epidemiological cut-off of 1 mg/L [44]. In subsequent updates of the same surveillance study including up to 3499 *C. difficile* isolates, a fidaxomicin MIC ≥ 4 mg/L was observed only in a single case [45,46]. Low MIC_90_ values for fidaxomicin were also displayed by *C. difficile* isolates from phase-2 (38 isolates, MIC_90_ of 0.125 mg/L, range ≤ 0.008 to 0.25 mg/L) and phase-3 (719 isolates, MIC_90_ of 0.25 mg/L, range 0.03 to 1 mg/L) studies of fidaxomicin, with only one strain isolated from a patient from a phase-3 study who developed rCDI showing a fidaxomicin MIC of 16 mg/L at the time of rCDI [47,48].

According to the in vitro studies reported above, reduced susceptibility to fidaxomicin is very rare; although, it has seldom been described [49]. In vitro, reduced susceptibility to fidaxomicin was selected through serial passages in a medium over a range of drug concentrations [50]. *C. difficile* isolates with reduced fidaxomicin susceptibility selected through serial passages harbored mutations in *rpoB*, encoding the β-subunit of RNA polymerase, or in *CD22120*, encoding a homolog of the family of transcriptional regulators MarR [50,51]. In a subsequent study, three *C. difficile* mutants with reduced susceptibility to fidaxomicin (MIC of 2, 8, and >32 mg/L, respectively) after the introduction of non-synonymous single-nucleotide polymorphisms in *rpoB* by allelic exchange also showed attenuated growth and reduced sporulation capacity, toxin A/B production, and cytotoxicity compared with the parental strain [52]. In a hamster model, the three mutants had impaired virulence in comparison to the parental strain; although, caecum colonization capacity was similar to the parental strain [52]. In a more recent study, a V1143D mutation was characterized in the *rpoB* gene of a clinical *C. difficile* isolate with fidaxomicin MIC > 64 mg/L and was associated with a less marked fitness defect than previously reported [53].

Regarding other particular characteristics, fidaxomicin and its metabolite OP-1118, differently from vancomycin, are able to inhibit sporulation (spore formation) of *C. difficile*, a fact which is thought to contribute to the observed increased rates of sustained response and reduced risk of recurrence in comparisons with other treatments (see the following section), since spores may persist after completion of a successful treatment course and subsequently germinate and proliferate, leading to a novel CDI episode [54,55,56]. After spores are formed, fidaxomicin, like vancomycin, is unable to inhibit germination, but both agents are able to counteract the outgrowth of vegetative cells from germinating spores [57]. However, fidaxomicin, but not vancomycin, has been demonstrated to persist on *C. difficile* spores after washing in saline and fecal filtrate, with consequent higher inhibitory effect on the outgrowth of vegetative cells and toxin production [58,59]. A substantial direct inhibitory effect of fidaxomicin and OP-1118 on toxin production may also explain the less frequent detection of post-treatment toxin production in fidaxomicin-treated patients than in vancomycin-treated patients [56,60,61]. A reduction in toxin A- and toxin B-mediated inflammatory responses and colonic tissue damage has also been described following exposure to fidaxomicin [62,63]. Another effect of fidaxomicin reported in in vitro studies, not observed with vancomycin, is the inhibition of biofilm formation, which could have implications for reducing the risk of both *C. difficile* colonization and CDI [64,65,66,67]. Finally, reduced shedding and environmental contamination by *C. difficile* have been described with fidaxomicin treatment more than with metronidazole or, although to a lesser extent, vancomycin [68,69,70,71].

## 4. Results of Phase-3/4 Randomized Controlled Trials

A summary of the main results of phase-3/4 RCTs assessing the efficacy of fidaxomicin for the treatment of CDI in adult patients is available in Table 1.

The first two large phase-3 randomized controlled trials (RCTs) assessing the efficacy of fidaxomicin for the treatment of CDI were the OPT-80-003 and OPT-80-004 studies [72,73]. Of note, patients with life-threatening or fulminant CDI were excluded from these studies [72,73]. In the non-inferiority, double-blind OPT-80-003 RCT, fidaxomicin (200 mg orally twice daily for 10 days) was compared to vancomycin (125 mg orally four times daily for 10 days) for the treatment of CDI [73]. The primary endpoint was clinical cure (defined as resolution of the symptoms and no need for further CDI treatment), assessed on the second day after the end of treatment. The secondary endpoints were rCDI (defined as diarrhea plus toxin test positivity on stool within 4 weeks after treatment of the previous episodes) and global cure (defined as clinical cure plus lack of rCDI). The primary study populations were the modified intention to treat (mITT) population (patients with documented CDI who received at least one dose of the study drug) and the per-protocol population (patients of the mITT population who received at least 3 days of treatment in the case of failure and at least 8 days of treatment in the case of clinical cure). Regarding the primary endpoint, fidaxomicin was found to be noninferior to vancomycin in terms of clinical cure both in the mITT population (88.2% (253/287) vs. 85.8% (265/309) in the fidaxomicin and vancomycin arms, respectively; lower margin of the 97.5% confidence interval [CI] for difference equal to −3.1%) and in the per-protocol population (92.1% (244/265) vs. 89.8% (254/283) in the fidaxomicin and vancomycin arms, respectively; lower margin of the 97.5% CI for difference equal to −2.6%). Regarding secondary endpoints, a lower frequency of rCDI was observed in fidaxomicin-treated than vancomycin-treated patients, both in the mITT population (15.4% (39/253) vs. 25.3% (67/265) in the fidaxomicin and vancomycin arms, respectively, with 95% CI for the difference from −16.6% to −2.9%) and in the per-protocol population (13.3% (28/211) vs. 24.0% (53/221) in the fidaxomicin and vancomycin arms, respectively, with 95% CI for the difference from −17.9% to −3.3%). A reduced frequency of rCDI in fidaxomicin-treated than in vancomycin-treated patients was retained in most subgroups; although, not in the subgroup of patients with CDI due to the 027 ribotype (27.1% (16/59) vs. 20.9% (14/67) in the fidaxomicin and vancomycin arms, respectively, in the mITT population). Finally, a higher frequency of global cure was registered in fidaxomicin-treated than in vancomycin-treated patients, both in the mITT population (74.6% (214/287) vs. 64.1% (198/309) in the fidaxomicin and vancomycin arms, respectively, with 95% CI from 3.1% to 17.7%) and in the per-protocol population (77.7% (206/265) vs. 67.1% (190/283) in the fidaxomicin and vancomycin arms, respectively, with 95% CI for the difference from 3.1% to 17.9%) [73]. Dosing schedules, endpoints, and primary study populations of the OPT-80-004 study were defined as in the OPT-80-003 study [72,73]. With regard to clinical cure (primary endpoint), fidaxomicin achieved noninferiority to vancomycin also in the OPT-80-004 study, both in the mITT population (87.7% (221/252) vs. 86.8% (223/257) in the fidaxomicin and vancomycin arms, respectively; lower margin of the 97.5% CI for difference equal to −4.9%) and in the per-protocol population (91.7% (198/216) vs. 90.6% (213/235) in the fidaxomicin and vancomycin arms, respectively; lower margin of the 97.5% CI for difference equal to −4.3%). A lower frequency of rCDI was observed in fidaxomicin-treated than in vancomycin-treated patients also in the OPT-80-004 study, both in the mITT population (12.7% (28/221) vs. 26.9% (60/223) in the fidaxomicin and vancomycin arms, respectively, with 95% CI for the difference from −21.4% to −6.8%) and in the per-protocol population (12.8% (23/180) vs. 25.3% (46/182) in the fidaxomicin and vancomycin arms, respectively, with 95% CI for the difference from −20.3% to −4.4%). Differently from the OTP-80-003 study, in the OTP-80-004 study, a lower frequency of rCDI in the fidaxomicin arm was also registered in the subgroup of patients with CDI due to the 027 ribotype (22.2% (12/54) vs. 38.0% (19/50) in the fidaxomicin and vancomycin arms, respectively, in the mITT population). As in the OTP-80-003 study, a higher frequency of global cure (also defined as a sustained response) was registered in fidaxomicin-treated than in vancomycin-treated patients, both in the mITT population (76.6% (193/252) vs. 63.4% (163/257) in the fidaxomicin and vancomycin arms, respectively, with 95% CI from 5.2% to 20.9%) and in the per-protocol population (79.6% (172/216) vs. 65.5% (154/235) in the fidaxomicin and vancomycin arms, respectively, with 95% CI for the difference from 5.9% to 22.1%) [72].

Different meta-analyses were conducted by pooling data from OTP-80-003 and OTP-80-004 after the two RCTs were released. In one of them, an exploratory, post hoc time-to-event analysis was conducted by means of fixed-effect meta-analysis and Cox regression [74]. Overall, the analysis included 1164 patients (ITT population) from the two RCTs and showed a reduction of persistent diarrhea, rCDI, or death (composite endpoint) of 40% (95% CI from 26% to 51%) through day 40 in fidaxomicin-treated patients vs. vancomycin-treated patients [74]. In another meta-analysis pooling data from the two RCTs, the odds ratio (OR) for clinical cure was 1.17 for fidaxomicin vs. vancomycin as reference (95% CI from 0.82 to 1.66) [75]. In subgroup analyses, the OR for clinical cure was 1.45 (95% CI from 0.63 to 3.36) and 0.86 (95% CI from 0.50 to 1.47) in patients with non-severe CDI and severe CDI, respectively. The OR for rCDI was 0.47 for fidaxomicin vs. vancomycin as a reference (95% CI from 0.34 to 0.65). In subgroup analyses, the OR for rCDI was 0.49 (95% CI from 0.32 to 0.74) and 0.46 (95% CI from 0.26 to 0.79) in patients with non-severe CDI and severe CDI, respectively. The OR for global cure was 1.75 for fidaxomicin vs. vancomycin as a reference (95% CI from 1.35 to 2.27). In subgroup analyses, the pooled OR for global cure was 1.92 (95% CI from 1.37 to 2.69) and 1.49 (95% CI from 0.99 to 2.26) in patients with non-severe CDI and severe CDI, respectively [75]. In another meta-analysis with pooled data from OTP-80-003 and OTP-80-004, the OR for symptomatic cure (defined as initial resolution of diarrhea and no evidence of recurrence up to 4 weeks) was 1.17 for fidaxomicin vs. vancomycin as reference (95% CI from 1.07 to 1.27) [76].

Combining data from the OTP-80-003 and OTP-80-004 studies, the efficacy of fidaxomicin vs. vancomycin for the treatment of CDI was evaluated in the following subgroups of patients with CDI: (i) patients who were concomitantly receiving other antibiotics for concomitant infections; and (ii) patients who were not receiving other concomitant antibiotics [77]. In presence of other concomitant antibiotic treatments, the clinical cure was higher in fidaxomicin-treated than in vancomycin-treated patients (90.0% (81/90) vs. 79.4% (81/102) in the fidaxomicin and vancomycin arms, respectively, with 95% CI for the difference from 0.2% to 20.4%), whereas clinical cure was similar between the two arms in the absence of other concomitant antibiotic treatments (92.3% (361/391) vs. 92.8% (386/416) in the fidaxomicin and vancomycin arms, respectively, with 95% CI for the difference from −4.1% to 3.2%). With regard to secondary endpoints, rates of rCDI and global cure were lower and higher, respectively, in fidaxomicin-treated than in vancomycin-treated patients both in patients receiving concomitant antibiotics and in patients not receiving concomitant antibiotics, in line with the main results of OTP-80-003 and OTP-80-004 [77]. Another exploratory post hoc analysis of combined data from OTP-80-003 and OTP-80-004 was conducted in the subgroups of CDI patients with and without cancer [78]. In patients with cancer, the clinical cure was 85.1% (74/87) and 74.0% (71/96) in patients treated with fidaxomicin and in patients treated with vancomycin, respectively (OR 2.00, with 95% CI from 0.95 to 4.22). In patients without cancer, the clinical cure was 88.5% (400/452) and 88.7% (417/470) in patients treated with fidaxomicin and in patients treated with vancomycin, respectively (odds ratio (OR) 0.98, with 95% CI from 0.65 to 1.47). The rates of rCDI and global cure were lower and higher, respectively, in fidaxomicin-treated than in vancomycin-treated patients both in patients with cancer and in patients without cancer, again in line with the main results of OTP-80-003 and OTP-80-004. Of note, the median time to resolution of diarrhea was longer in patients with cancer than in those without cancer in the vancomycin arm (123 h vs. 58 h, log-rank *p* < 0.001), but not in the fidaxomicin arm (74 h vs. 54 h, log-rank *p* = 0.145) [78]. Another study combining data from OTP-80-003 and OTP-80-004 employed restriction endonuclease analysis (REA) typing on paired isolates from the index episode and recurrence (available from 90/146 patients with rCDI), to differentiate between relapse (identical REA type strain) and reinfection (different REA type strain) [79]. There was no comparison between fidaxomicin and vancomycin in terms of the study endpoints of the original RCTs, whereas a comparison between the two agents was made in terms of mean time to relapse and reinfection. The mean time to relapse in fidaxomicin-treated and in vancomycin-treated patients was 11.2 days (standard deviation (SD) ±6.1) and 14.3 days (SD ±6.2), respectively (*t* test, *p* = 0.044). The mean time to reinfection in fidaxomicin-treated and in vancomycin-treated patients was 13.9 days (SD ±7.5) and 16.8 days (SD ±4.6), respectively (*t* test, *p* = 0.497) [79]. In a further study combining data from OTP-80-003 and OTP-80-004 and employing whole-genome sequencing for distinguishing relapse (paired samples from CDI and rCDI ≤ 2 single-nucleotide variants apart) from reinfection (paired samples from CDI and rCDI > 2 single-nucleotide variants apart), the reduction in the risks of relapse and reinfection in fidaxomicin-treated vs. vancomycin-treated patients was explored using competing risk models (subdistribution hazard ratio (sHR) 0.40 for relapse, with 95% CI from 0.25 to 0.66; sHR 0.33 for reinfection, with 95% CI from 0.11 to 1.01) [80]. Regarding patients with rCDI, their possible differential risk of developing further recurrences based on fidaxomicin vs. vancomycin treatment was explored in a subset analysis of combined data from the OTP-80-003 and OTP-80-004 studies, including 128 patients who had a recent CDI episode before the index episode leading to enrollment [81]. In this analysis, the frequency of clinical cure was similar in fidaxomicin-treated and vancomycin-treated patients (93.7% (74/79) vs. 91.6% (76/83) in the fidaxomicin and vancomycin arms, respectively), whereas the frequency of rCDI (in this subgroup representing a second occurrence of rCDI) was lower in fidaxomicin-treated than in vancomycin-treated patients (19.7% (13/66) vs. 35.5% (22/62) in the fidaxomicin and vancomycin arms, respectively, with 95% CI for the difference from −30.4% to −0.3%) [81]. Finally, treatment with fidaxomicin was associated with a 60% reduced risk of recurrence in comparison with vancomycin in a logistic regression model adjusted for *C. difficile* strain, age, and concomitant antibiotics in 567 patients from OTP-80-003 and OTP-80-004 studies [82].

Subsequently, a phase-3 study was also conducted in Japan to assess the efficacy of fidaxomicin vs. vancomycin for the treatment of CDI. The drugs were administered at the same dosages of OTP-80-003 and OTP-80-004 [83]. The primary endpoint was global cure, which was assessed in the full analysis set (FAS) population and achieved in 67.3% (70/104) and 65.7% (71/108) of fidaxomicin-treated and vancomycin-treated patients, respectively (95% CI for the difference from −11.3 to 13.7, thereby not allowing demonstration of noninferiority). In a post hoc analysis of FAS patients who received at least 3 days of treatment, the global cure was 72.2% (70/97) and 67.0% (71/106) in fidaxomicin-treated and vancomycin-treated patients, respectively (95% CI for the difference from −7.9% to 17.1%). The frequency of rCDI in the FAS for recurrence (FAS-R) population, composed of FAS patients who achieved clinical cure during the index episode, was 19.5% (17/87) and 25.3% (24/95) in fidaxomicin-treated and vancomycin-treated patients, respectively (95% CI for the difference from −16.7% to 7.0%) [83]. In a network meta-analysis including pooled data from the RCT conducted in Japan, the OTP-80-003 RCT, and the OTP-80-004 RCT, the clinical cure was found to be similar between fidaxomicin and vancomycin (OR 1.17 with vancomycin as a reference, with 95% credible intervals from 0.78 to 1.48), whereas fidaxomicin showed a favorable association both with rCDI (OR 0.50 with vancomycin as a reference, with 95% credible intervals from 0.37 to 0.68) and global cure (OR 1.61 with vancomycin as a reference, with 95% credible intervals from 1.27 to 2.05) [84].

Three other small RCTs assessing the efficacy of fidaxomicin at standard dosage (200 mg twice daily for 10 days) for the treatment of CDI were recently published [61,85,86]. In one of them, the standard dosage of fidaxomicin was compared with vancomycin (125 mg four times daily for 10 days) for the treatment of first CDI episodes [85]. The primary endpoint was the percentage of subjects achieving a reduction of at least 2 log_10_ colony-forming units (CFU)/g of spores in stools from screening to the end of therapy, and was achieved more frequently in fidaxomicin-treated than vancomycin-treated patients (67% (8/12) vs. 14% (1/7)) [85]. In another small, pilot RCT of 12 patients, the standard dosage of fidaxomicin was compared with vancomycin (125 mg four times daily for 10 days) with respect to the reduction in toxin concentrations in stools from baseline, with results suggesting a favorable association between fidaxomicin and a sustained reduction in toxins A and B up to day 30 after therapy [61]. Finally, 64 patients with rCDI were randomized into three arms (standard dosage fidaxomicin, standard dosage vancomycin, and fecal microbiota transplant (FMT)) in a third small RCT [86]. The primary endpoint was a combination of clinical resolution and a negative toxin polymerase chain reaction at 8 weeks after allocation, and was achieved in 33% (8/24), 19% (3/16), and 71% (17/24) of patients receiving fidaxomicin, vancomycin, and FMT, respectively [86]. A recent meta-analysis pooling data also from these three small RCTs in addition to OTP-80-003, OTP-80-004, and the RCT conducted in Japan, showed a comparable clinical cure between fidaxomicin and vancomycin (risk ratio (RR) 1.02, with 95% CI from 0.98 to 1.06), and favorable associations between fidaxomicin and reduced risk of rCDI (RR 0.59, with 95% CI from 0.47 to 0.75) and improved global cure (RR 1.18, with 95% CI from 1.09 to 1.26) [87].

The results of the EXTEND RCT were published in 2018 [88]. EXTEND was an open-label phase-3b/4 RCT comparing extended-pulsed fidaxomicin (administered orally at 200 mg twice daily on days 1–5, and then only once daily on alternate days from day 7 to day 25) vs. vancomycin (at the standard dosage of 125 mg four times daily for 10 days) in inpatients aged 60 years or older. The primary endpoint was sustained clinical cure at 30 days after the end of treatment in the modified FAS population (all randomized patients who received at least one dose of the study drug), and was achieved in 70% (124/177) and 59% (106/179) of patients in extended-pulsed fidaxomicin and vancomycin arms, respectively (95% CI for the difference from 1.0% to 20.7%), thereby demonstrating superiority (a fact which is in line with the enhanced suppression of *C. difficile* by a pulsed fidaxomicin regimen in preclinical studies [89]; although, the limitations of the lack of comparison vs. the standard fidaxomicin dosage and an extended-pulsed vancomycin regimen were acknowledged in the EXTEND study). With regard to rCDI (one of the study’s secondary endpoints), lower rates of recurrences were registered in the extended-pulsed fidaxomicin arm than in the vancomycin arm at day 40 (3/124 (2.4%) vs. 22/125 (17.6%)), day55 (7/124 (5.6%) vs. 23/125 (18.4%)), and day 90 (11/124 (8.8%) vs. 23/125 (18.4%)) [88]. Of note, pharmacokinetic/pharmacodynamic data from patients enrolled in EXTEND revealed that fidaxomicin concentrations in stools were above the MIC_90_ of *C. difficile* isolates (inferred from in vitro studies) until day 26 ± 1 [90]. The subgroup analyses of the EXTEND study showed higher clinical cure rates in the extended-pulsed fidaxomicin arm independent of age, prior CDI, infection with PCR-ribotype 027, CDI severity, or presence of cancer [91]. A post hoc analysis of the EXTEND study conducted after testing stools of enrolled patients at screening, also with the BioFire FilmArray Gastrointestinal Panel (BioMérieux, Basingstoke, UK), suggested that co-infection with other pathogens could possibly explain clinical failures [92]. In a meta-analysis pooling data from five of the RCTs discussed above plus the EXTEND study, fidaxomicin was associated with improved sustained symptomatic cure compared to vancomycin (OR 0.67, with 95% CI from 0.55 to 0.82) [93].

In hematopoietic stem cell transplantation (HSCT) recipients, the development of CDI is more frequent than in the general population of hospitalized patients, and it has been associated with an increased risk of bloodstream infections, new-onset graft versus host disease, and non-relapse mortality [94,95,96,97]. In the double-blind DEFLECT-1 RCT, fidaxomicin was compared to a placebo for the prophylaxis of CDI in HSCT recipients (either allo-HSCT or auto-HSCT) undergoing fluoroquinolone prophylaxis [98]. The primary composite endpoint, assessed in the mITT population (subjects receiving at least one dose of study drug/placebo) was prophylaxis failure, defined as confirmed CDI, receipt of anti-*C. difficile* drugs for any indication, or missed assessment of CDI for any reason. Fidaxomicin was administered at the dosage of 200 mg daily, starting from 2 days after conditioning or initiation of prophylaxis with fluoroquinolones, and continuing until 7 days after neutrophil engraftment or completion of prophylaxis with fluoroquinolones/treatment with other antimicrobials, for up to 40 days. Prophylaxis failure was similar in patients receiving fidaxomicin and in patients receiving placebo (28.6% (86/301) vs. 30.8% (92/299), respectively, with 95% CI for the difference from −5.1% to 9.5%); although, it is of note that most failures occurred because of non-CDI events and confirmed CDI was less frequent in the fidaxomicin arm than in the placebo arm in a sensitivity analysis (4.3% (13/301) vs. 10.7% (32/299), with 95% CI for the difference from 2.2% to 10.6%) [98].

Among currently ongoing RCT comparing fidaxomicin vs. other treatments for CDI is OpTION, a double-blind study that is being conducted in patients with rCDI and is comparing the efficacy of three different treatment regimens: (i) 200 mg of fidaxomicin twice daily, for 10 days; (ii) 125 mg of vancomycin four times daily, for 10 days; and (iii) 125 mg of vancomycin four times daily, for 10 days, followed by a taper/pulse regimen of vancomycin for 3 weeks [99]. Other ongoing phase-3/4 RCTs are comparing fidaxomicin vs. FMT in patients with rCDI (NCT05266807, NCT05201079). The results of an open-label RCT comparing standard dosage fidaxomicin vs. standard dosage vancomycin in patients with CDI receiving concurrent antibiotics for other infections have been recently released, with the primary endpoint of clinical cure having been registered in 73% (54/74) and 62.9% (44/70) of patients in the fidaxomicin and vancomycin arms, respectively [100]. Among secondary endpoints, rCDI developed in 3.3% (2/60) and 4.0% (2/50) of patients in the fidaxomicin and vancomycin arms, respectively [100].

In RCTs, fidaxomicin was overall well tolerated. In the OPT-80-003 and OPT-80-004 studies, its safety profile was similar to oral vancomycin, and there were no differences between the two drugs in the frequency of death or serious adverse events [101]. The only numerical imbalances in these studies were related to gastrointestinal hemorrhage (4.1% vs. 3.1% in fidaxomicin-treated and vancomycin-treated patients, respectively) and leukopenia (4.1% vs. 1.7% in fidaxomicin-treated and vancomycin-treated patients, respectively); although, there was no evidence of a causal relationship between fidaxomicin administration and the occurrence of these events [101]. Similar tolerability profiles of fidaxomicin and vancomycin were observed in the phase-3 study conducted in Japan [83]. The incidence of treatment-emergent adverse events was similar in the extended-pulsed fidaxomicin arm and in the vancomycin arm in the EXTEND study [88]. One death in the vancomycin arm was deemed as being related to the study drug by the investigators [88]. The registered drug-related adverse events were 15% and 20% in the fidaxomicin and placebo arms in the DEFLECT-1 RCT [98].

## 5. Conclusions

In both the recently updated IDSA/SHEA guidelines and the updated ESCMID guidance document, fidaxomicin is preferentially recommended as first-line treatment over vancomycin both for the first episode of CDI and for rCDI (see Table 2 for more details) [11,12]. Although vancomycin remains a suitable alternative to fidaxomicin (noninferiority was indeed the rule in phase-3 RCTs with regard to the primary endpoint of clinical response), for shaping these recommendations particular attention was devoted to the improved global cure and reduced risk of rCDI observed with fidaxomicin compared to vancomycin in RCTs. The overall scenario is thus shifting from “administer vancomycin first, because of reduced cost and similar efficacy” to “consider fidaxomicin first, in view of the global benefits for the patient, if feasible”. With regard to feasibility, fidaxomicin still remains more costly than vancomycin, and, while the major driver of choice should solidly remain the global benefit for the patient, consideration of available resources should also be necessarily weighed in the balance. Against this background, a clear mistake would be that of continuing to administer vancomycin for any first CDI episode only because of reduced costs, thereby ignoring the evidence arising from RCTs about the improved global benefits following fidaxomicin treatment. Rather, risk models for rCDI should be used for selecting patients to preferentially receive fidaxomicin (i.e., to clearly identify those patients for whom fidaxomicin-driven global benefits are relevant). In our opinion, precisely stratifying risk groups for rCDI will represent a crucial research trajectory of future real-life studies on the treatment of initial CDI episodes. In addition, after reviewing the results of existing RCTs summarized in the previous sections, we also consider some other remaining grey areas as relevant fields for current and future research: (i) the exact positioning of the extended-pulsed fidaxomicin regimen, and its comparative efficacy with an extended-pulsed vancomycin regimen; (ii) the comparative efficacy of fidaxomicin vs. vancomycin in severe and severe-complicated CDI; (iii) the efficacy of fidaxomicin plus bezlotoxumab in preventing rCDI in comparison to other bezlotoxumab-including regimens; and (iv) when to precisely consider FMT instead of treatment with oral drugs, including fidaxomicin. Elucidating all of these remaining areas could further optimize the current positioning of fidaxomicin within CDI and rCDI treatment algorithms and, in turn, patients’ health.

## Figures and Tables

**Table 1:** Main efficacy data from phase-3/4 randomized controlled trials of fidaxomicin for the treatment of CDI in adult patients.

Author, Year	Fidaxomicin	Comparator/s	Study Population	Frequency	% Difference ^a^ (95% CI)
Study Name [Ref]	Regimen	(Dosage)	Endpoint (Primary/Secondary)	(Events/Treated)	
	(Dosage)				
Louie et al., 2011 OTP-80-003 [73]	Standard regimen (200 mg orally twice daily for 10 days)	Vancomycin (125 mg orally four times daily for 10 days)	*mITT population* ^b^		
			*Clinical cure (primary)*		
			Fidaxomicin	88.2% (253/287)	2.4 (−3.1 ^d^)
			Vancomycin	85.8% (265/309)	Reference
			*rCDI (secondary)*		
			Fidaxomicin	15.4% (39/253)	−9.9 (−16.6 to −2.9)
			Vancomycin	25.3% (67/265)	Reference
			*Global cure (secondary)*		
			Fidaxomicin	74.6% (214/287)	10.5 (3.1 to 17.7)
			Vancomycin	64.1% (198/309)	Reference
			*Per-protocol population* ^c^		
			*Clinical cure (primary)*		
			Fidaxomicin	92.1% (244/265)	2.3 (−2.6 ^d^)
			Vancomycin	89.8% (254/283)	Reference
			*rCDI (secondary)*		
			Fidaxomicin	13.3% (28/211)	−10.7 (−17.9 to −3.3)
			Vancomycin	24.0% (53/221)	Reference
			*Global cure (secondary)*		
			Fidaxomicin	77.7% (206/265)	10.6 (3.1 to 17.9)
			Vancomycin	67.1% (190/283)	Reference
Cornely et al., 2012 OTP-80-004 [72]	Standard regimen (200 mg orally twice daily for 10 days)	Vancomycin (125 mg orally four times daily for 10 days)	*mITT population* ^b^		
			*Clinical cure (primary)*		
			Fidaxomicin	87.7% (221/252)	0.9 (−4.9 ^d^)
			Vancomycin	86.8% (223/257)	Reference
			*rCDI (secondary)*		
			Fidaxomicin	12.7% (28/221)	−14.2 (−21.4 to −6.8)
			Vancomycin	26.9% (60/223)	Reference
			*Sustained response (secondary)*		
			Fidaxomicin	76.6% (193/252)	13.2 (5.3 to 21.0)
			Vancomycin	63.4% (163/257)	Reference
			*Per-protocol population* ^c^		
			*Clinical cure (primary)*		
			Fidaxomicin	91.7% (198/216)	1.1 (−4.3 ^d^)
			Vancomycin	90.6% (213/235)	Reference
			*rCDI (secondary)*		
			Fidaxomicin	12.8% (23/180)	−12.5 (−20.5 to −4.5)
			Vancomycin	25.3% (46/182)	Reference
			*Global cure (secondary)*		
			Fidaxomicin	79.6% (172/216)	14.1 (6.0 to 22.2)
			Vancomycin	65.5% (154/235)	Reference
Mikamo et al., 2018 [83]	Standard regimen (200 mg orally twice daily for 10 days)	Vancomycin (125 mg orally four times daily for 10 days)	*FAS population*		
			*Global cure (primary)*		
			Fidaxomicin	67.3% (70/104)	1.2 (−11.3 to 13.7)
			Vancomycin	65.7% (71/108)	Reference
			*FAS-R population* ^e^		
			*rCDI (secondary)*		
			Fidaxomicin	19.5% (17/87)	−4.9 (−16.7 to 7.0)
			Vancomycin	25.3% (24/95)	Reference
Housman et al., 2016 [85]	Standard regimen (200 mg orally twice daily for 10 days)	Vancomycin (125 mg orally four times daily for 10 days)	*Patients with CDI*		
			*Reduction of spores (primary)* ^f^		
			Fidaxomicin	66.7% (8/12)	52.4 (NA)
			Vancomycin	14.3% (1/7)	Reference
Hvas et al., 2019 [86]	Standard regimen (200 mg orally twice daily for 10 days)	Vancomycin (125 mg orally four times daily for 10 days) or FMT	*Patients with rCDI*		
			*Clinical resolution (primary)* ^g^		
			Fidaxomicin	33.3% (8/24)	14.5% (NA)
			FMT	70.8% (17/24)	52.0% NA)
			Vancomycin	18.8% (3/16)	Reference
Guery et al., 2018 EXTEND [88]	Extended-pulsed regimen (200 mg twice daily on days 1–5, and then only once daily on alternate days from day 7 to day 25)	Vancomycin (125 mg orally four times daily for 10 days)	*Modified FAS population* ^h^		
			*Sustained clinical cure (primary)*		
			Fidaxomicin	70.1% (124/177)	OR 1.62 (1.04 to 2.54)
			Vancomycin	59.2% (106/179)	Reference
			*Per-protocol population*		
			*rCDI at day 40 (secondary)*		
			Fidaxomicin	2.4% (3/124)	OR 0.12 (0.04 to 0.41)
			Vancomycin	17.6% (22/125)	Reference
			*rCDI at day 55 (secondary)*		
			Fidaxomicin	5.6% (7/124)	OR 0.31 (0.13 to 0.73)
			Vancomycin	18.4% (23/125)	Reference
			*rCDI at day 90 (secondary)*		
			Fidaxomicin	8.8% (11/124)	OR 0.49 (0.23 to 1.04)
			Vancomycin	18.4% (23/125)	Reference

CDI, *Clostridioides difficile* infection; CI, confidence interval; FAS, full analysis set; FMT, fecal microbiota transplant; mITT, modified intention-to-treat; NA, not available; OR, odds ratio; rCDI, recurrent CDI. ^a^ Unless otherwise indicated. ^b^ Including patients with documented CDI who received at least one dose of study drug. ^c^ Including patients of the mITT population who received at least 3 days of treatment in the case of failure and at least 8 days of treatment in the case of clinical cure. ^d^ One-sided 97.5% CI. ^e^ FAS patients who achieved clinical cure during the index episode. ^f^ Defined as percentage of subjects achieving a reduction of at least 2 log_10_ colony-forming units (CFU)/g of spores in stools from screening to the end of therapy. ^g^ Defined as combination of clinical resolution and a negative toxin polymerase chain reaction at 8 weeks after allocation. ^h^ Including all randomized patients who received at least one dose of study drug.

**Table 2:** Current IDSA/SHEA and ESCMID recommendations regarding fidaxomicin for the treatment of CDI and rCDI.

Guidelines/Guidance Document	Recommended Treatment for First CDI Episode *	Recommended Treatment for rCDI *
ESCMID guidance document [12]	The use of a standard regimen of fidaxomicin is suggested over vancomycin *(Strong recommendation, with moderate level of evidence)* Risk stratification should be considered when access to fidaxomicin is limited (e.g., older age >65 years plus one or more of the following: healthcare-associated CDI; hospitalization; in the previous 3 months, administration of concomitant antibiotics, initiation of PPIs during or after diagnosis of CDI; previous CDI episode) *(Good practice statement)* When fidaxomicin is unavailable or unfeasible, vancomycin is a suitable alternative *(Strong recommendation, with high level of evidence)* An extended-pulsed regiment of fidaxomicin could be considered in case of risk of rCDI, especially in old inpatients *(Weak recommendation, with low level of evidence)* For severe or severe-complicated CDI, a standard regimen of fidaxomicin or vancomycin is suggested *(good practice statement)*	If the initial CDI episode was treated with metronidazole or vancomycin, the use of a standard regimen of fidaxomicin is preferentially recommended *(Strong recommendation, with low level of evidence)* If the initial CDI episode was treated with fidaxomicin, considered bezlotoxumab in addition to fidaxomicin *(Weak recommendation, with moderate level of evidence; “addition to fidaxomicin” as a good practice statement)* When fidaxomicin and bezlotoxumab are unavailable or unfeasible, consider a tapered/pulsed regimen of vancomycin *(Weak recommendation, with very low level of evidence)* For multiple recurrences, FMT or bezlotoxumab in addition to standard of care is suggested *(Weak recommendation, with moderate level of evidence for FMT and low level of evidence for bezlotoxumab)*
IDSA/SHEA guidelines [11]	The use of a standard regimen of fidaxomicin is suggested over a standard course of vancomycin. A high value is placed on the beneficial effects and the safety of fidaxomicin, with implementations depending on available resources and with vancomycin remaining an acceptable alternative *(Conditional recommendation with moderate certainty of evidence)*	The use of a standard or extended-pulsed regimen of fidaxomicin is suggested over a standard regimen of vancomycin. For a first rCDI episode, vancomycin in a standard or tapered/pulsed regimen is an acceptable alternative. For multiple recurrences, possible options are fidaxomicin (standard or extended-pulsed regimen), vancomycin in a tapered/pulsed regimen, vancomycin followed by rifaximin, and FMT *(Conditional recommendation with low certainty of evidence)*

CDI, *Clostridioides difficile* infection; ESCMID, European Society of Clinical Microbiology and Infectious Diseases; FMT, fecal microbiota transplant; IDSA, Infectious Diseases Society of America; PPIs, proton pump inhibitors; rCDI, recurrent CDI; SHEA, Society for Healthcare Epidemiology of America. * For other recommendations about the use of other agents (e.g., bezlotoxumab) or FMT and not directly involving a decision about fidaxomicin please refer to the original guidelines/guidance documents [11,12]. For a fulminant CDI episode (hypotension or shock, ileus, or megacolon), IDSA/SHEA guidelines recommend oral/nasogastric tube vancomycin 500 mg four times daily plus intravenous metronidazole 500 mg thrice daily plus rectal instillation of vancomycin if ileus.

## Data Availability

Not applicable.
